# Decreased Expression of CD14 in MSU-Mediated Inflammation May Be Associated with Spontaneous Remission of Acute Gout

**DOI:** 10.1155/2019/7143241

**Published:** 2019-06-13

**Authors:** Lihua Duan, Jiao Luo, Qiang Fu, Ke Shang, Yingying Wei, Youlian Wang, Yan Li, Jie Chen

**Affiliations:** ^1^Department of Rheumatology and Clinical Immunology, Jiangxi Provincial People's Hospital, Nanchang, China; ^2^Department of Rheumatology and Clinical Immunology, University of Lübeck, Germany; ^3^Department of Rheumatology and Clinical Immunology, The First Affiliated Hospital of Xiamen University, Xiamen, China; ^4^Department of Scientific Research and Education, Jiangxi Provincial People's Hospital, Nanchang, China

## Abstract

Gout is a common metabolic disease in humans, and it is due to persistently elevated levels of uric acid in the blood. At high levels, uric acid crystallizes and the crystals deposit in joints and surrounding tissues, resulting in an attack of gout. Interestingly, the gout attack can spontaneously resolve within a few days. However, the self-limited mechanism of gout remains elusive. It has been demonstrated that CD14 plays an important role in self-remission of gout. In this study, we found that the proportion of CD14-positive PBMCs was decreased in gout patients when compared with healthy controls and the serum sCD14 level was also considerably decreased in gout patients in comparison to healthy controls. In addition, sCD14 levels were positively correlated with CRP levels. Furthermore, the effect of MSU on the levels of CD14 in healthy volunteer's PBMC was explored in in vitro experiment. The results showed that CD14 expression on macrophage and sCD14 levels in the culture supernatants were significantly decreased after MSU treatment. However, there was no significance in the levels of membrane CD14 and sCD14 in healthy volunteer's PBMC stimulated by LPS. Taken together, these results suggest that CD14 might play an important role in self-remission of gout.

## 1. Introduction

Gout is a common metabolic disease in humans, which is caused by the purine metabolism disorder [1]. The pathological feature of gout is chronic deposition of monosodium urate (MSU) crystals in joints and surrounding tissues, which results from serum uric acid concentration rise above the physiological saturation [2] [3]. The deposition of MSU in joints and surrounding tissues activates the resident tissue macrophage, leading to an acute inflammatory response [4]. The activated macrophages produce abundant amounts of TNF-*α*, IL-1*β*, IL-6, IL-8, and chemotactic factors. Recent studies have demonstrated that IL-1*β* is the critical cytokine in the development of MSU-induced inflammation [5, 6]. However, IL-1*β* indirectly recruits a marked number of neutrophils into the joint cavity through promoting the production of adherence molecules of endothelial cells [7, 8]. This process is associated with the clinical manifestation of an acute gout attack. Interestingly, acute gout attack is an acute inflammatory disease characterized by self-limiting inflammation in response to the deposition of MSU crystals in the joints or tissues [9]. Until recently, the reason for the spontaneous rapid resolution of inflammation in gout was still unclear.

CD14, a GPI-anchored protein, is constitutively expressed on the surface of various cells, including monocytes, macrophages, polymorphonuclear neutrophils [10], B cells [11], and dendritic cells [12]. CD14 is the specific coreceptor for lipopolysaccharide (LPS) which is a compound of the outer cell wall of Gram-negative bacteria [13]. CD14 has two forms, membrane-bound (mCD14) and a circulating soluble (sCD14) [14, 15]. On the cell surface of monocyte, LPS interacts with mCD14 and the LPS-binding protein (LBP) and forms a high-affinity trimolecular complex which result in the intracellular signaling pathway activation and lots of inflammatory cytokines, including IL-1*β* and IL-6 [16–18]. sCD14 is the form of CD14 without glycosylphosphatidylinositol tail and can be detected in serum [14, 19]. It was demonstrated that sCD14 is released from cellular membrane CD14 through the protease-dependent mechanism [14, 20]. Actually, sCD14 also play an important role in LPS-mediated activation of cells that lack membrane-bound CD14 such as endothelial and epithelial vascular [21, 22].

Previous studies have shown that MSU crystals activate macrophage in a manner that requires the canonical signaling pathway via TLR4 and TLR2. In addition, TLR2 and TLR4 mediate macrophage uptake of MSU crystals in vitro. Furthermore, the TLR signaling pathway recruits the intracellular adapter protein myeloid differentiation factor (MyD88), which also plays a critical role in the MSU uptake and NF-*κ*B activation [23, 24]. As a pattern molecule, CD14 is also crucial for MSU crystal uptake, caspase-1 activation, and IL-1*β* production. A decreased uptake of MSU was observed in CD14 knockout mice [25]. However, the acute attack of gout is usually self-limited, and the reason for the spontaneous rapid resolution of MSU-induced inflammation was rather enigmatic. Some studies showed that a switch from proinflammatory to anti-inflammatory macrophages and increased production of anti-inflammatory mediators TGF-*β* and IL-10 have been discussed as potential factors responsible for the resolution of inflammation in gout [26]. Recently, neutrophil extracellular trap formation (NETosis) was also involved in the self-limited inflammation. It is highly potent to trap and cleave MSU-induced inflammatory cytokines within minutes [27]. In this study, we demonstrated that CD14 might play an important role in self-remission of gout.

## 2. Materials and Methods

### 2.1. Patients' Characteristics and Controls

40 adult patients (2 females, aged 57.25 ± 19.62 years) with a diagnosis of gout based on the American College of Rheumatology/European League Against Rheumatism criteria [28] were consecutively enrolled in the study after providing informed consent. All patients were referred from the Department of Rheumatology and Clinical Immunology at the Jiangxi Provincial People's Hospital and the First Affiliated Hospital of Xiamen University. The control group consisted of 31 healthy volunteers (3 females, aged 56.75 ± 12.36 years) that were enrolled after giving informed consent. Additional blood for PBMC isolation was harvested from 26 patients and 20 controls.  Table 1 depicts the demographic and clinical characteristics of all patients and controls. All clinical manifestations and laboratory findings were recorded on the day of blood withdrawal.

### 2.2. Flow Cytometry Analysis

The peripheral blood of gout patients and healthy controls was collected in coagulant tubes. Peripheral blood mononuclear cells (PBMCs) were isolated by standard Ficoll-Hypaque density gradient centrifugation for 30 min. PBMC was stained by anti-human CD14 or CD68 (BioLegend).

### 2.3. ELISA

Four milliliters of blood was collected in sterile coagulant tubes and was then centrifuged at 3,500 rpm for 5 min at ambient temperature to obtain serum, which was immediately frozen and stored at -80°C until batch analysis. Concentrations of sCD14 were determined by ELISA kits (R&D) according to the manufacturer's protocols.

### 2.4. Cell Culture

PBMCs of healthy volunteers were stimulated with 30 *μ*g, 100 *μ*g, and 300 *μ*g/mL of MSU for 16 h. As a contrast, PBMCs from healthy volunteers were stimulated with 100 ng, 1 *μ*g, and 10 *μ*g/mL of LPS for 16 h. The cells and culture supernatants were harvested for flow cytometry and ELISA analysis, respectively.

### 2.5. Statistical Analysis

All data were analyzed using GraphPad Prism 5. Statistical significance was determined by unpaired Student's *t*-test, Mann-Whitney *U* test, and Spearman's correlation analysis which were used to calculate significance.

## 3. Results

### 3.1. Clinical Characteristics of Gout Patients

The clinical characteristics of gout patients (Table 1) were summarized for this study. 40 patients with gout and 31 healthy controls from Southern Chinese population were enrolled. The mean age for gout patients was 57.25 ± 15.62 years, and there were 38 males and 2 females. The mean ± SD of CRP, ESR, and UA is 75.21 ± 70.29 mg/L, 51.76 ± 29 mm/h, and 492.80 ± 148.3 *μ*mol/L, respectively. Among these 40 patients, 35 patients (87.5%) had acute gout attack, 15 patients (37.5%) had tophi, 13 patients (32.5%) had gouty kidney damage, and 4 patients (10%) had fever. It was remarkable that 27 patients (67.5%) had hyperuricemia.

### 3.2. Decreased CD14 Expression in Gout Patients

**Figure 1:**
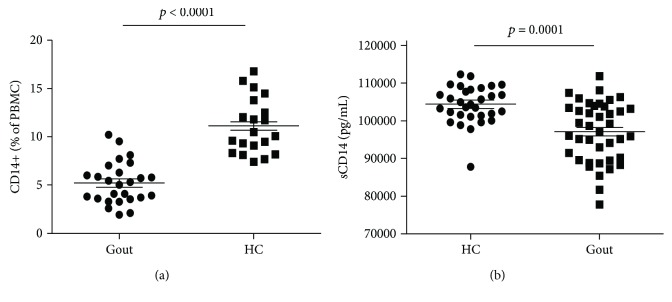
The CD14 expression was decreased in gout patient. (a) The PBMCs from gout patients (*n* = 26) and healthy controls (HC) (*n* = 20) were harvested and stained with the anti-CD14 antibody and then subjected to flow cytometry to detect the mCD14 expression. The CD14-positive cells were gated for analysis. (b) The serum levels of sCD14 in gout patients (*n* = 40) and HC (*n* = 31) were detected by ELISA. The Mann-Whitney *U* test was conducted to evaluate the significant difference of patients and controls. Each symbol represents an individual sample; horizontal lines indicate median values.

To explore the role of CD14 in the spontaneous remission of gout, PBMC and serum were harvested to analyze the membrane and soluble CD14 in the gout patients and healthy controls by flow cytometry and ELISA. In comparison with healthy controls, the expression of mCD14 on PBMC was significantly lower in gout patients (Figure 1(a)). Consistently, sCD14 in serum from gout patients was also markedly lower than that from healthy controls (Figure 1(b)).

### 3.3. Correlation between sCD14 in Serum and Laboratory Parameters in Gout Patients

**Figure 2:**
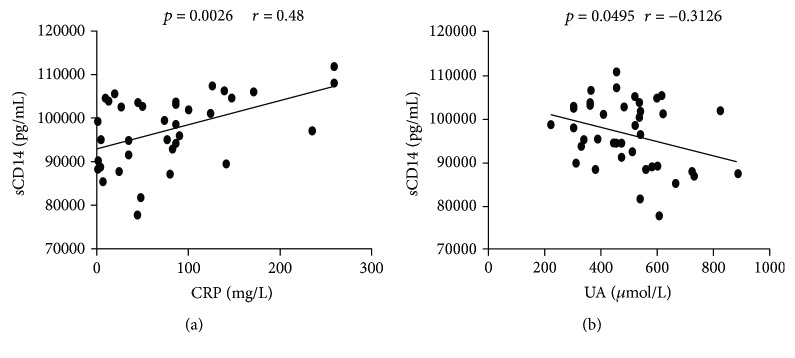
The correlation of sCD14 with inflammatory marker CRP and uric acid in gout patients. The relationships between sCD14 levels and CRP and UA in gout patients were analyzed. (a) sCD14 was positively correlated with CRP in gout patients (*r* = 0.48, *p* = 0.0026). (b) sCD14 was negatively correlated with UA in gout patients (*r* = −0.3126, *p* = 0.0495). Spearman's correlation analysis was used to evaluate significance.

C-reactive protein (CRP) is an acute phase and inflammatory marker for the disease activity index in patients with gout. To evaluate the role of CD14 in the development of acute gout, the relationship between sCD14 levels and inflammatory marker CRP in gout patients was analyzed. As shown in Figure 2(a), we found that the sCD14 levels were positively correlated with CRP levels (*r* = 0.48, *p* = 0.0026). However, no significantly negative correlation was observed between serum sCD14 and UA (*r* = −0.3126, *p* = 0.0495). In keeping with previous study, our result pinpointed that CD14 was also linked to the development of gout inflammation. In addition, it is worth noting that the decreased CD14 in the gout patients might be implicated in the process of resolution of inflammation in gout.

### 3.4. MSU Downregulates Membrane CD14 Expression on PBMCs

**Figure 3:**
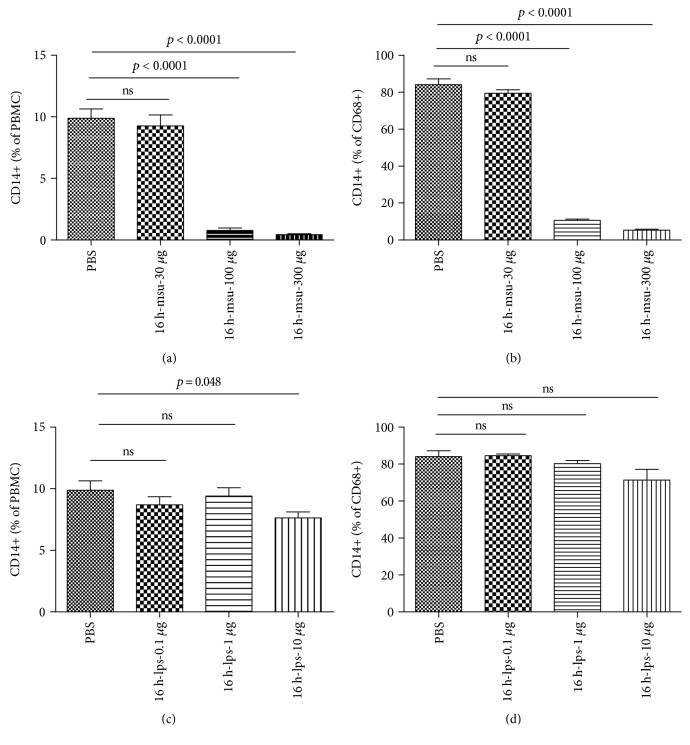
MSU directly reduced mCD14 expression on PBMCs. (a, b) PBMCs were harvested from healthy volunteers and stimulated with with PBS, MSU (30 *μ*g/mL, 100 *μ*g/mL, and 300 *μ*g/mL), or LPS (0.1 *μ*g/mL, 1 *μ*g/mL, and 10 *μ*g/mL) for 16 h. CD14 expression on PBMCs was detected by flow cytometry (a, c), and the CD68-positive cells were also gated to analyze the CD14 expression by flow cytometry (b, d). Data from three independent experiments are presented as mean. The unpaired Student *t*-test was used to calculate the significance.

To further ascertain that the CD14 expression was downregulated by MSU stimulation, we harvested the PBMCs from healthy controls and treated them with different doses of MSU. Although low MSU concentration has no obvious effect on the mCD14 expression of PBMCs, a significantly reduced CD14 expression was observed in PBMCs stimulated with high MSU concentration (Figure 3(a)). Next, CD68, the specific marker of monocytes/macrophage, were labeled and subjected to flow cytometry to analyze the CD14 expression. In parallel with the above result, the CD14 expressions on CD68+ monocytes/macrophages were markedly reduced in PBMCs with higher MSU concentration treatment (Figure 3(b)). However, LPS has no obvious effect on CD14 expression in PBMC and CD68+ monocytes/macrophages (Figures 3(c) and 3(d)).

### 3.5. MSU Reduces the Level of Soluble CD14

**Figure 4:**
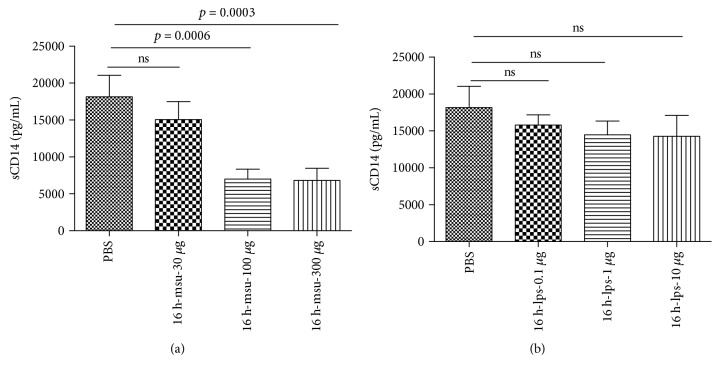
Decreased sCD14 in supernatants of PBMCs stimulated with MSU treatment. PBMCs from healthy volunteers were stimulated with PBS, MSU (30 *μ*g/mL, 100 *μ*g/mL, and 300 *μ*g/mL) (a), and LPS (0.1 *μ*g/mL, 1 *μ*g/mL, and 10 *μ*g/mL) (b). After 16 h, the culture supernatants were harvested and subjected to ELISA analysis. Data from three independent experiments are presented as mean ± SD. The unpaired Student *t*-test was used to calculate the significance.

Next, we tested the level of sCD14 in the culture supernatant of PBMCs treated with different concentrations of MSU or LPS. Consistently, sCD14 levels on the PBMCs with PBS and 30 *μ*g/mL MSU treatment were not reduced. However, a significantly decreased sCD14 production was observed in PBMC stimulated with 100 *μ*g/mL MSU (*p* = 0.0006) and 300 *μ*g/mL MSU (*p* = 0.0003) (Figure 4(a)). In keeping with mCD14 expression, the sCD14 productions in the culture supernatant of PBMC stimulated with LPS (0.1 *μ*g/mL, 1 *μ*g/mL, and 10 *μ*g/mL) were not reduced (Figure 4(b)). Therefore, our data suggested that MSU reduced the CD14 production during the development of MSU-induced inflammation.

## 4. Discussion

In the present study, we demonstrated the CD14 expression and its potential role in the process of spontaneous remission of acute gout. mCD14 expression was significantly decreased on the PBMCs from gout patients when compared with those from healthy control subjects. In addition, the serum sCD14 level was also considerably decreased in gout patients. Furthermore, we found that MSU directly reduced the CD14 expression in the PBMCs from healthy volunteers. However, there is no significant effect in mCD14 and sCD14 production by PBMCs which were treated with LPS. Previous studies have shown the critical role of CD14 in the development of acute gout; we also observed a positive correlation between sCD14 and CRP levels. Therefore, the reduced CD14 production in MSU-induced inflammation might contribute to spontaneous rapid resolution of inflammation.

It is well known that many mechanisms contribute to the process of inflammatory resolution [29]. The reason for the spontaneous rapid resolution of inflammation in gout was still unclear. It has been demonstrated that macrophages clear the cellular apoptotic remnants to help stop the inflammatory cascade through upregulating the anti-inflammatory cytokines, such as TGF-*β* and IL-10 [26]. Similarly, phagocytosis of crystals not only initiates the acute gout attack but also makes it possible to evolve into spontaneous resolution of acute gout attack by producing TGF-*β* and IL-10 [30]. These anti-inflammatory cytokines play an important role in inhibiting the inflammatory process, including inhibition of the receptor expression on the surface of leukocytes [26]. Furthermore, numerous research also demonstrated that the pathologic potential of MSU can be modified by proteins and other agents, which might be the explanation for why crystals are not always inflammatory [31, 32]. Recently, neutrophil extracellular trap formation (NETosis) was also involved in the self-limited inflammation. It is highly potent to trap and cleave MSU-induced inflammatory cytokines within minutes [27]. In this study, we found that CD14 was positively correlated with the inflammatory marker CRP. The glucocorticoid (GC) treatment was often used in controlling sepsis, which was associated with the downregulation of CD14 production in monocytes and macrophage [33]. Because MSU crystals taken by phagocytes were dependent on TLR4, TLR2, and CD14 [23–25], glucocorticoid was often used to restrain the inflammatory response induced by MSU [34]. Although CD14 was important for the development of gout, we observed a decreased CD14 production in the gout patients. It indicated that CD14 might contribute to spontaneous rapid resolution of inflammation.

Previous study showed that acute gouty inflammation is triggered by cellular recognition of the naked MSU crystal which was dependent on CD14 [25], and the detection of monocytes and macrophages having ingested MSU crystals is the gold standard clinical procedure to identify gout. However, the phagocytosis of crystals will promote naive macrophages to a mature type, which can produce lots of anti-inflammatory cytokine TGF-*β*. Consistently, noninflammatory deglutition of MSU crystals was observed in the mature macrophages and macrophage cell lines in vitro [35]. Therefore, blockage of MSU ingestion by downregulating CD14 expression might be a potential therapy for acute gout attack. Furthermore, some studies also showed that the self-limitation of gout can be induced by phagocytosis of crystals by macrophages, leading to the suppression of cellular inflammatory signaling pathway activation. Our study here demonstrated that both mCD14 and sCD14 expressions were decreased in gout patients when compared with healthy control subjects. Interestingly, mCD14 can be cleaved into sCD14 form, which can be served as a macrophage activity marker. The serum sCD14 levels were increased in patients with multiple sclerosis [36], and sCD14 is thought to be an early diagnostic and prognostic marker for patients with systemic infections [37]. However, sCD14 was decreased in MSU-induced inflammation in our study. These data provide a novelty mechanism in the regulation of CD14 expression, and further study should be conducted to certify the mechanism of decreased CD14 production in the development of gout.

In conclusion, these results suggested that CD14 might play a vital role in the mechanism of spontaneous remission of gout. The understanding of the detailed mechanism will provide us a novel opportunity to interfere with inflammation induced by MSU. Certainly, further studies are required to explore the specific regulation mechanism between CD14 and gout.

## Figures and Tables

**Table 1:** Demographic data and clinical characteristics of subjects in the study.

	Gout patients (*n* = 40)	Healthy controls (*n* = 31)
Age (year, mean ± SD)	57.25 ± 15.62	56.75 ± 12.36
Male sex (%)	95%	92%
CRP (C-reactive protein) (mg/L, mean ± SD)	75.21 ± 70.29	—
UA (uric acid) (*μ*mol/L, mean ± SD)	492.80 ± 148.3	—
ESR (erythrocyte sedimentation rate) (mm/h, mean ± SD)	51.76 ± 29.00	—
Acute phase (%)	87.5%	—
Hyperuricemia (%)	67.5%	—
Tophi (%)	37.5%	—
Gouty kidney damage (%)	32.5%	—
Fever (%)	10%	—

## Data Availability

The data used to support the findings of this study are available from the corresponding authors upon request.
